# Unravelling the Role of HSP70 as the Unexplored Molecular Target in Age-Related Macular Degeneration

**DOI:** 10.7759/cureus.8960

**Published:** 2020-07-02

**Authors:** Rajat Kumar, Ravi Soni, Stacey E Heindl, Dwayne A Wiltshire, Safeera Khan

**Affiliations:** 1 Ophthalmology, California Institute of Behavioral Neurosciences and Psychology, Fairfield, USA; 2 Neurology, California Institute of Behavioral Neurosciences and Psychology, Fairfield, USA; 3 Medicine, California Institute of Behavioral Neurosciences and Psychology, Fairfield, USA; 4 Internal Medicine, California Institute of Behavioral Neurosciences and Psychology, Fairfield, USA

**Keywords:** age-related macular degeneration, heat shock protein 70, apoptosis, inflammation, oxidative stress, hne, retinal pigment epithelial cell

## Abstract

The motive behind writing this paper was to highlight the relationship between heat shock protein 70 (HSP70) and age-related macular degeneration (AMD) to explore the potential role of HSP70 as a molecular target in AMD therapy. We performed a comprehensive literature search in various databases and finally found 43 relevant studies related to our objective. In our research, we found that in AMD, oxidative stress causes increased inflammation and excessive apoptosis due to the accumulation of aberrant proteins in retinal pigment epithelium (RPE) cells. The long-lasting overstimulation of the defence system leads to RPE degeneration and results in visual impairment or vision loss. However, after thorough research, it was found that HSP70's role as an immunomodulator, the guardian of the proteolytic pathway and regulator of apoptosis makes it a potential therapeutic target in AMD.

## Introduction and background

Jane, a 68-year-old white woman, a chronic smoker, was struggling while reading a newspaper under dim light due to occasional distortion of the lines. She called it a side effect of ageing and did not consult a doctor. A few years later, Jane realized while reading her favourite novel, the page centre had become hazy, which frightened her. The very next day, she approached an ophthalmologist. On questioning, she gave a history of her father losing vision in the right eye in a similar manner. After a thorough assessment, the doctor diagnosed her with non-neovascular age-related macular degeneration (AMD). However, despite the accurate diagnosis, Jane faced difficulties in day-to-day activities for the rest of her life due to the lack of effective treatment [1].

Just like Jane, approximately 9% of people suffer from this disease globally [2]. AMD is the primary cause of irreversible loss of vision and blindness among the elderly [2]. It is a progressive, chronic, neurodegenerative disease influencing the macula, which plays an essential role in central and sharp vision in the retina, thereby severely affecting daily necessary activities like reading, dialling numbers and recognizing faces [3]. Pathologically AMD involves degradation of the macular retinal pigment epithelial (RPE) cells, Bruch's membrane, and choriocapillaris. When patients come to the clinic at an early stage, the first sign observed by an ophthalmologist is the presence of yellow deposits of protein aggregates called drusen, found between RPE and posteriorly located Bruch's membrane. Due to ineffective treatment or late diagnosis, AMD progresses to late-stage AMD. The advanced stage of AMD falls into two categories: neovascular/wet/exudative AMD and non-neovascular/dry/non-exudative AMD [4]. In wet AMD, there is increased vascular permeability and proliferation of atypical vessels, known as choroidal neovascularisation (CNV), in the choroid. CNV can extend through gaps in Bruch's membrane and cause subretinal fluid exudation, lipid deposition, and detachment of the RPE from the choroid [5]. In neovascular AMD, patients are likely to develop sudden and severe central vision loss within days or weeks. However, in dry AMD, there is progressive atrophy of RPE over a large area, also called geographic atrophy, which is accompanied by a significant vision impairment advancing gradually and insidiously over months to years [6]. In the past, numerous studies on the retina have allowed ophthalmologists to develop strategies to treat AMD. Currently, the most commonly used method to treat AMD is anti-angiogenic vascular endothelial growth factor (VEGF) inhibitors like ranibizumab and bevacizumab. They have shown improvement in central vision in approximately 30% of patients [7]. Still, the long-term treatment of VEGF inhibitors causes geographic atrophy and subretinal fibrosis in a majority of the cases [8]. Till now, none of the therapies has shown effective results in clinical trials due to the lack of an ideal molecular target [7].

Before finding out the much-needed molecular target, it is crucial to understand the triad of AMD pathogenesis: oxidative stress, inflammatory insult, and aberrant apoptosis. Physiologically, reactive oxygen species (ROS) play significant positive roles in bacterial infections, signal transduction, and other cellular functions [9]. However, elevated levels of ROS production cause oxidative stress, abnormally increase the immune response, and overstimulate intrinsic apoptosis. Inflammation, in response to stress, activates a wide range of inflammatory cells to eliminate toxic substances and promote the viability of RPE via apoptosis. But, under prolonged stress, chronic inflammation causes massive tissue destruction resulting in the development of AMD [10]. In response to increased cellular stress, RPE stimulates the heat shock transcription factor HSF1, which facilitates the expression of HSP70, an endogenous protein well known for its cytoprotective role. Unfortunately, with an increase in age, the functioning capacity of HSF1 declines which impairs the ability of senescent RPE cells to cope up with ongoing stress [11]. The effect of HSP70 on the triad of AMD is poorly understood. It is estimated that the global impact of AMD will be 196 million in 2020 and will increase to around 300 million by 2040, yet we are far from developing a cure [2]. Therefore, to help people like Jane to live a healthy and independent life, we attempt to investigate the importance of HSP70 in combating AMD-induced cellular insult and support the idea of utilizing HSP70 as a novel potential molecular target in macular degeneration.

## Review

RPE cells battle every day against cellular insults to perform their vital functions in the retina. They endure an intense amount of oxidative stress due to excessive consumption of oxygen, products from outer segments of photoreceptors undergoing lipid peroxidation, and continuous light exposure. These cells have an inbuilt mechanism to tackle such cellular insults, but due to ageing and chronic stress exposure, cellular homeostasis is disturbed. Long-lasting exposure to oxidative stress results in the accumulation of proteins, cytotoxic inflammatory response, and widespread RPE degeneration due to increased aberrant programmed-cellular deaths [12]. Although extensive research has been done on AMD, no molecular target has effectively neutralized the toxic effect of the triad of AMD pathogenesis.

The end of apoptotic degeneration

Under normal conditions, apoptosis is a part of the standard immune-defence mechanism of RPE cells in response to cellular stress. However, abnormal induction of apoptosis by ROS causes an increase in cell death and retinal tissue damage, leading to retinal atrophy and vision loss in AMD patients [13]. Caspase, a group of aspartate-specific cysteine proteases, mediates cellular apoptosis. In RPE, the accumulation of misfolded proteins acts as a stressor and activates the caspase-mediated mitochondrial apoptotic pathway (intrinsic apoptosis). The aggregated proteins are perceived as danger signals and result in activation of pro-apoptosis factors, such as p53, Bax, Bid, Akt, Apaf-1, and other Bcl-2 families. The pro-apoptotic elements open the permeability transition pore (PTP) to alter mitochondrial membrane permeability. The change in permeability triggers the release of cytochrome c, a pro-apoptotic factor, into the cytosol to form a complex with apoptotic protease activating factor-1 (Apaf-1). This newly built complex, also called the apoptosome, recruits and activates pro-caspase-9. The activation of caspase-9 further activates and releases the executioner caspase-3, which aids in cellular degradation of RPE cells [14]. HSP70 plays a crucial role in the regulation of apoptosis by suppressing apoptotic pathways via inhibition of pro-apoptosis factors. The detailed mechanism behind the anti-apoptotic function of HSP70 is still unclear. Research evidence suggests that HSP70 links itself to Apaf-1 in an ATPase-dependent manner, subsequently preventing the formation of apoptosome. This leads to inhibition of the intrinsic apoptotic pathway. Also, the study proposed that association of HSP70 with a co-chaperone, HSP40, blocks the translocation of Bax from mitochondria to cytosol and suppresses apoptosis induced by nitric oxide. This provides further evidence of the anti-apoptotic role of HSP70 [15]. Besides inhibiting pro-apoptosis factors, HSP70 shields proteins that inhibit apoptosis, such as XIAP, to protect RPE [16]. Yang et al. demonstrated that the end product of oxidative stress, 4-hydroxynonenal (HNE), can inhibit the protective role of HSP70 on XIAP by modifying HSP70's spatial structure in a concentration-dependent manner [17]. Hence, with an increase in oxidative stress, there is a possibility of increased apoptosis due to HSP70 modification by HNE. Also, the long-lasting stress on RPE, at a certain point in time, surpasses the cytoprotective functioning limit of HSP70, leading to rising oxidative damage. Therefore, the delivery of exogenous HSP70 is needed to combat the accumulated stress and to limit the progress of the disease.

HSP70, the guardian of protein homeostasis

HSP70, the protector of the proteolytic pathway, regulates the repair of oxidized proteins and prevents their aggregation by forming essential associations with proteasome and lysosomes in protein degradation [18]. HSP70-induced protein homeostasis mainly involves chaperone-mediated autophagy (CMA) and proteasomal disintegration. Currently, not much is known about these two mechanisms.

Some proteins possess a specific sequence which acts as a signal and translocates them from the cytoplasm to lysosomal lumen for degradation. This cytosolic degradation is mediated by CMA [19]. The excess accumulation of misfolded protein aggregates results in CMA activation, which helps in the elimination of distorted proteins as part of the cellular protein-homeostasis systems. The cytosolic HSP70, one of the molecular chaperones in the lysosomal lumen, induces the unfolding of the target protein before its transportation across the lysosomal membrane. It facilitates its translocation by pulling the target protein inside the lysosome via ATPase-dependent entropic pulling for degradation [20]. At one end, the chaperone binds to the target protein, which possesses a peptide sequence that includes the KFERQ motif. On the other side, it associates with lysosome-associated membrane protein 2A (LAMP-2A), a receptor present on the cytosolic lysosomal surface. This association guides the transport of misfolded protein across the lysosomal membrane [19,20]. However, HSP70 is not the only chaperone involved in CMA. Numerous other chaperone proteins either regulate the activity of HSP70 or act as chaperone themselves, such as heat shock protein 40 (HSP40), heat shock protein 90 (HSP90), HSP70 interacting protein (HIP), and HSP70-HSP90 organizer protein (HOP). The co-chaperone HSP40 stimulates the ATPase activity of HSP70, whereas HSP90 helps in the refolding of misfolded proteins and prevents the unfolded proteins from aggregating. Moreover, HIP and HOP play vital roles in CMA by stimulating the assembly of the complex and linking HSP70 with HSP90, respectively [21].

HSP70 prevents the formation of protein aggregates by suppressing the formation of abnormal proteins. During translation, the folding of the emerging nascent proteins from the ribosome is delayed by HSP70 until the essential sequence which folds the entire domain is exposed at the ribosomal surface. Consequently, the freshly synthesized polypeptide is released. Under normal conditions, the proposed fate of the new polypeptide is automatic folding of the protein or transfer to other chaperones like HSP90 for further folding [22]. However, when HSP90 chaperone is impaired, the substrate either re-attaches itself to HSP70 or undergoes proteasomal degradation. The sequential polypeptides bound to HSP70 are tagged by ubiquitin with the help of the co-chaperone CHIP (E3 ubiquitin-protein ligase). Subsequently, the ubiquitylated substrate is targeted for degradation by the proteasome [23]. With time, the increase in cellular insults hinders the endogenous protein quality control pathway, leading to the accumulation of ubiquitin-tagged proteins. The ubiquitinated proteins induce stimulation of HSF1 and subsequent expression of HSP70 [24]. HSP70 associates with HSP40 with the aid of the J domain to form a complex that targets aggregated stress-induced protein misfolding due to the collapse of proteasome machinery [25]. HSP40 facilitates the delivery of misfolded protein to HSP70 and stimulates the ATPase activity of the same to generate a force needed to break open aggregates and release the trapped polypeptides via entropic pulling. The exact mechanism of entropic pulling in protein disaggregation is currently not precise [25,26]. Nevertheless, HSP70 has a practical limit; once it is exceeded, protein aggregates are formed, which are transferred to juxtanuclear location, subsequently forming aggresomes [27]. These aggresomes accumulate inside the lysosome as lipofuscin, causing lysosomal damage. Also, these aggregates collect in extracellular space between the RPE and Bruch's membrane in the form of drusen, the earliest clinical sign of AMD [28]. The overexpression of HSP70 can enhance proteasome-mediated proteolysis and suppress the formation of aggresomes to prevent impairment of critical cellular functions.

HSP70 plays a double-edged role as an immunomodulator

In AMD pathogenesis, HSP70 majorly contributes to promoting cellular viability by suppressing apoptosis, playing the role of an antioxidant, and by subduing inflammatory response. The essential modulatory role of HSP70 in inflammation depends on its location [28]. The inflammatory cascade is activated by specialized pattern recognition receptors (PRRs) like Toll-like receptors (TLRs) and scavenger receptors found on immune cells. Chronic degenerative diseases like AMD are known for disrupting cellular homeostasis by inducing inflammation. In macular degeneration, abnormal protein formation is interpreted as a danger signal by endogenous danger-associated molecular pattern (DAMP). PRRs recognize and bind to these DAMP molecules. This association sets off the signal transduction to activate nuclear factor kappa B (NF-κB) [29]. The pro-inflammatory transcription factor, NF-κB, is usually present in an inactive state, attached to NF-κB inhibitor beta (IkB) in the cytosol. When DAMP attaches to the PRR, it subsequently activates IkB kinase (IkK), which triggers phosphorylation and degradation of IkB to activate NF-κB [30]. The activated NF-κB translocates to the nucleus of the immune cell and stimulates the pro-inflammatory transcription factors to instigate the expression of pro-inflammatory cytokines, such as IL-1β, IL-6, and TNF-α [31,32]. The role of HSP70 as an immunomodulatory protein was proposed in a study conducted by Yang et al., and it revealed that in ARPE-19 cells, the overexpression of HSP70 with the help of a co-inducer like arimoclomol significantly suppressed the production of pro-inflammatory cytokines which are commonly associated with AMD, such as IL-1β, IL-6 and TNF-α, whereas the levels of anti-inflammatory cytokines IL-10 and TGF-β1 were found to be increased [33,34]. The proposed mechanism utilized by HSP70 to reduce the production of pro-inflammatory cytokines, like TNF-α and IL-1β, is linked to inhibition of the activation of NF-κB by inhibiting IkK. The suppression of IkK weakens IkB's phosphorylation and proteasomal degradation, consequently impairing the translocation of NF-κB to the nucleus [35]. However, the role of HSP70 is not just limited to repressing stress-induced inflammation. A study by Yang et al. highlighted that exogenous injections of HSP70 significantly reduced the area of subretinal fibrosis in mice [36]. Several studies have been conducted to treat CNV in the wet form of AMD, which has led to the development of multiple therapeutic strategies, including verteporfin photodynamic therapy, and anti-VEGF therapy. However, very less is known about treating fibrotic changes in the foveal CNV lesion, which leads to severe, permanent visual impairment in patients with neovascular AMD [37]. Yang et al. showed that HSP70 is a potential molecular target to reduce subretinal fibrosis by increasing the production of IL-10. Further, this study provided evidence of TLR2/TLR4-dependent extracellular anti-inflammatory property of HSP70. Extracellular hsp70 enhanced the level of IL-10 without affecting IL-6 levels in mice having TLR2/TLR4 receptors. However, no significant rise was observed in mice without TLR2/TLR4 signalling [36].

According to another notion, pro-inflammatory mediators are induced by extracellular HSP70. A recent study showed that HNE, the end product of lipid peroxidation, did not affect the activity of intracellular HSP70. Still, it increased the extracellular efflux of HSP70 in a concentration-dependent manner, which resulted in a dramatic rise in pro-inflammatory cytokine levels [34]. The mechanism behind the transportation of HSP70, one of the 4-HNE targeted proteins, to extracellular space is poorly understood [33]. The author proposed that when HSP70 escapes into the extracellular space, it acts like DAMP molecule and associates with TLR2/TLR4 or scavenger receptors, leading to induction of inflammation by activating NF-κB [38]. The studies mentioned above talk about the correlation between HSP70 and NF-κB. Based on the location of HSP70, their relationship keeps changing. After going through the data, we collected, we are particular about the anti-inflammatory role of cytosolic protein, but the function of extracellular HSP70 is still controversial. Results of both Yang et al. and Yang et al. contradict with each other [34,36]. Whether extracellular protein association with TLR2/TLR4 is responsible for its anti-inflammatory or pro-inflammatory role is still unclear. Therefore, labelling HSP70 as a friend or a foe of inflamed RPE cells is not justified until further research is done on the extracellular and intracellular roles of HSP70.

Is HSP70 the ideal molecular target?

Till now, we have discussed how the expression of HSP70 can help in restraining enhanced inflammatory response, degradation of aggregated toxic proteins, and regulation of aberrant apoptosis. Conversely, without knowing the feasibility of therapeutic protein delivery, it cannot be deemed as an ideal molecular target. The efficacy of exogenously delivered HSP70 relies upon diffusion of the drug from the site of injection, quantitative uptake by RPE cells, and intracellular localization of the therapeutic protein. Researcher Subrizi et al. evaluated the dispersal of recombinant human HSP70 (rhHSP70) from the site of intravitreal injection on a pig eye. He observed that after the administration of radiolabeled, negatively charged rhHSP70, it immediately disseminated from negatively charged vitreous to the retina due to electrostatic repulsion between vitreous and HSP70. Utilizing the process of passive diffusion, HSP70 penetrated the multi-layered retina to reach the RPE. The results look promising. However, the involvement of the ex vivo model has its limitations. Ex vivo model excludes the age-related changes, for instance, impaired tightness of the intraocular barriers, contraction, and liquefaction of pathogenic vitreous state. The study further showed that less than 60% of the ARPE-19 cells internalized rhHSP70 with the help of flow cytometry. The possible reason behind less therapeutic uptake of the molecular target can be the formation of double layers by the differentiated ARPE-19 cells instead of forming monolayers. The upper layers had access to rhHSP70, but the bottom layer did not; therefore, cytometry showed false reduced uptake of the protein [28].

In the experiment, the internalized rhHSP70 accumulated in endosomes and lysosomes, which is an advantage for HSP70. HSP70 can easily eliminate the oxidized proteins accumulated in lysosomes, thereby preventing impairment of lysosomes [39]. Upregulation of HSP70 is possible by either inducing the expression of endogenous HSP70 or the delivery of rhHSP70. Numerous studies have been conducted to develop HSP70-induced therapeutic strategies for AMD. In this review, we will talk about the action of HSP70 co-inducers and rhHSP70 on oxidized RPE cells. A recent study demonstrated the active role of rhHSP70 in antagonizing oxidative cellular insult in RPE in vivo. ARPE-19 cells were exposed to hydrogen peroxide; subsequently, the results of cells treated with and without rhHSP70 were compared. The results showed that exogenous delivery of rhHSP70 reduced inflammation, improved cellular functions, and decreased the level of toxins, in comparison to the cells that did not receive such treatment. This evidence favours the antioxidant role of exogenous rhHSP70 [28].

In past decades, research studies have explored the potential role of HSP70 co-inducers, such as leucostatin, paeoniflorin, celastrol, and arimoclomol in the protection of health and function of RPE cells. These therapeutic protein co-inducers act by facilitating the transcriptional activation of a heat shock promoter, heat shock factor-1, and subsequently induce HSP70 expression [34,40,41]. Although the mechanism of HSP70 induction is the same, its effectiveness in combating oxidative stress is still uncertain. Moreover, evidence exists that ARPE-19 cells pre-treated by arimoclomol were more efficient in tackling HNE-induced stress than those treated with paeoniflorin. The difference between the two inducers is probably due to the difference in their HSP70 inducing capability. Arimoclomol acts on HSF-1 and increases the level of cytoplasmic HSP70, but the level of extracellular HSP70 remains unaffected, whereas drug paeoniflorin increases the level of both intracellular and extracellular HSP70. Paeoniflorin might have induced the pro-inflammatory property of extracellular HSP70 and decreased the anti-inflammatory effect of HSP70; therefore, it was less efficient in handling oxidative stress [34]. The reasoning behind the difference in arimoclomol and paeoniflorin HSP70 induction is not known; hence, further research should be done to understand the possible pathways of HSP70 induction by its co-inducers. Apart from drugs and rhHSP70, retinal laser therapies have shown induction of HSP70 in ARPE-19 in vivo model and experimental rabbit eyes. Although studies have been conducted on thermal laser treatment, more research needs to be done to define the damage threshold of thermal irradiation to induce maximal HSP70 with minimal lethal damage to the tissues [42,43].

**Figure 1:**
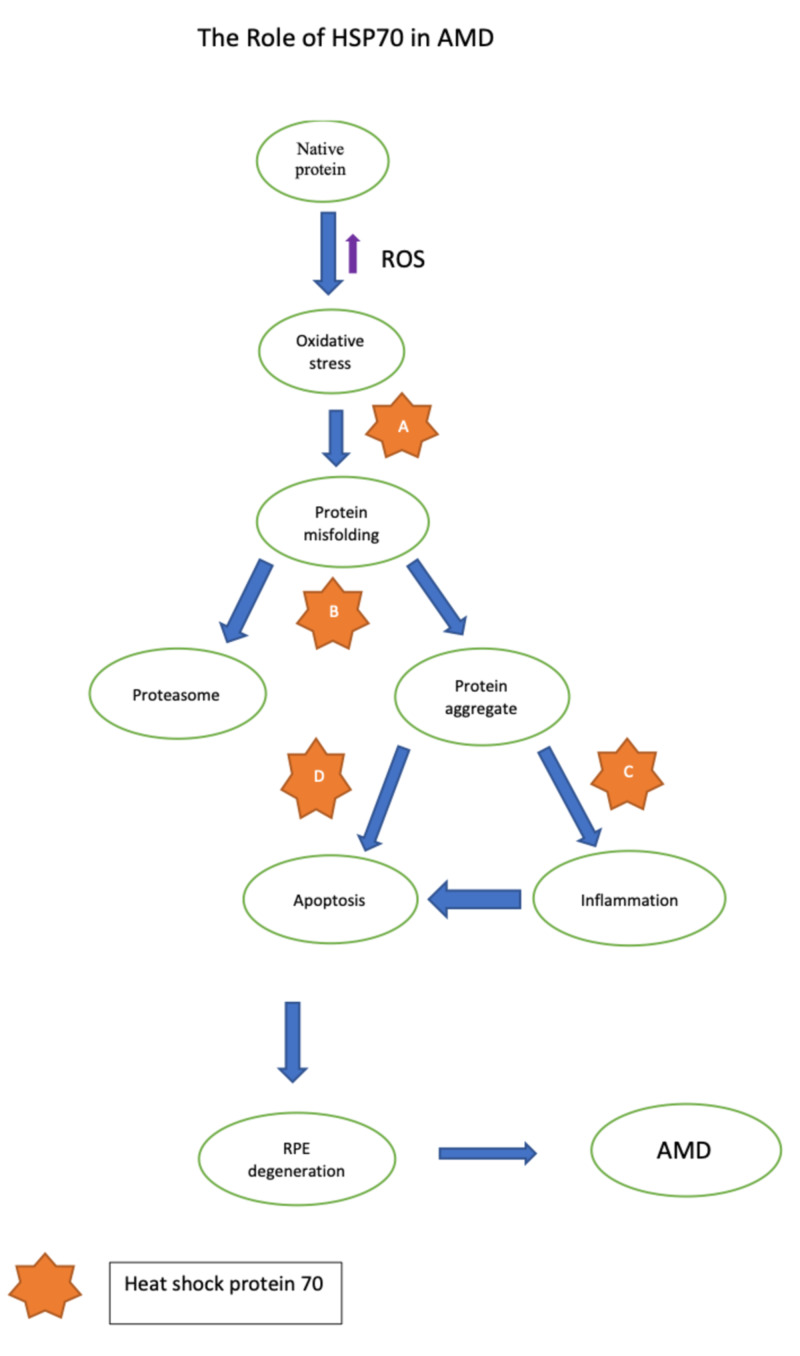
The role of HSP70 in AMD. (A) HSP70 prevents misfolding of native proteins in RPE cells which can decelerate the progression of the disease. (B) The misfolded proteins translocate to proteasome for degradation by HSP70. If proteasomal function is impaired, HSP70 machinery is stimulated and prevents the formation of protein aggregates. (C) The release of pro-inflammatory cytokines is inhibited HSP70 via inactivation of IkK. (D) HSP70 prevents aberrant apoptosis and RPE degeneration by impeding the release of pro-apoptotic factors. AMD: age-related macular degeneration, HSP70: heat shock protein 70, RPE: retinal pigment epithelium; ikK, IkB kinase

Based on the studies mentioned above, it is safe to propose that HSP70 has come close to fulfilling the criteria of an ideal therapeutic target (Figure 1), still limited by a lack of research on the application of heat shock protein (Table 1).

**Table 1:** Details of some of the studies from the review

Author’s Name	Year of Publication	Result	Conclusion
Lyu et al. [40]	2020	In canine retinal pigment epithelium (RPE) cells, arsenite-induced stress caused a significant increase in heat shock protein 70 (HSP70) expression. In addition, leucinostatin stimulated HSP70 expression by inducing heat shock factor-1.	This study suggests that leucinostatin enhances heat shock factor-1 to increase HSP70 expression in RPE cells.
McArdle et al. [11]	2019	The ability of a skeletal muscle to respond to an increase in reactive oxygen species (ROS) generation using an increased expression of HSP-like proteins decreases with age. This affects ROS homeostasis.	Advancing age disturbs the physiological functioning of ROS. In chronic cases, this results in neurodegenerative disorders.
Yang et al. [17]	2019	4-HNE induces late apoptosis in ARPE-19 cells, with increased levels of 4-HNE-modified HSP70 which decreased the levels of XIAP.	HSP70 can induce to apoptosis if it is modified by HNE.
Yang et al. [34]	2019	Intracellular HSP70 suppressed the production of pro-inflammatory cytokines. 4-HNE facilitated pro-inflammatory action in RPE cells by increasing the extracellular release of HSP70. However, when combined with efflux inhibitor methyl-β-cyclodextrin (MBC), the release of HSP70 was reduced and a decrease in levels of IL-6 was found.	HNE-induced modification of HSP70 antagonizes the anti-inflammatory role of HSP70 and boosts the level of IL-6. This effect can be blocked by administrating MBC.
Wang et al. [43]	2016	Results show that in non-damaging retinal therapy (NRT) the activation of protein can take place in a range of 25%-40%.	This study shows that there is a very narrow range of non-lethal activation of HSP70 by NRT.
Subrizi et al. [28]	2015	On exposing ARPE-19 cells with hydrogen peroxide, IL-6 production was reduced in cells treated with recombinant human HSP70 (rhHSP70), and also improved cell viability. rhHSP70 was localized in lysosomes.	Exogenously delivered HSP70 localizes in lysosomes and improves the viability of ARPE-19 on administration.
Yang et al. [36]	2013	Giving HSP70 exogenously, inhibited subretinal fibrosis in mice with Toll-like receptor (TLR)2/TLR4 and induced IL-10 but not in TLR2 and TLR4-deficient mice	Results suggest that exogenous HSP70 exhibits anti-inflammatory action by combining with TLR2/TLR4.
Ryhänen et al. [39]	2009	The study suggested that accumulation of lysosomes can be reversed. Also, it stated that autophagy aids in the clearance of deposits due to proteasomal inhibition.	After the failure of the proteasome-mediated pathway, autophagy is stimulated. It helps in clearing the toxic cellular material but excess stimulation can result in degradation of tissues.

## Conclusions

In our traditional review, we explained the potential role and controversies linked to the application of HSP70 as a therapeutic agent in treating AMD patients. The studies included in this review explain the cytoprotective role of HSP70 against the erratic natural defence system of the retina, which facilitates the progression of macular degeneration. Although studies have been conducted on exploring the relationship between HSP and AMD, no human clinical trials were found to determine its efficacy in the clinical scenario. This review lays the foundation for future research studies to explore HSP70 as the molecular target of age-related macular disease.

## References

[1] (2020). Centers for Disease Control and Prevention. Learn about age-related macular degeneration. https://www.cdc.gov/features/healthyvisionmonth/index.html.

[2] Wong WL, Su X, Li X, Cheung CMG, Klein R, Cheng CY, Wong TY (2014). Global prevalence of age-related macular degeneration and disease burden projection for 2020 And 2040: a systematic review and meta-analysis. Lancet Glob Health.

[3] Lim LS, Mitchell P, Seddon JM, Holz FG, Wong TY (2012). Age-related macular degeneration. Lancet.

[4] Cheung LK, Eaton A (2013). Age-related macular degeneration. Pharmacotherapy.

[5] De Jong PTVM (2006). Age-related macular degeneration. N Engl J Med.

[6] Sunness JS, Rubin GS, Applegate CA, Bressler NM, Marsh MJ, Hawkins BS, Haselwood D (1997). Visual function abnormalities and prognosis in eyes with age-related geographic atrophy of the macula and good visual acuity. Ophthalmology.

[7] Ambati J, Atkinson JP, Gelfand BD (2013). Immunology of age-related macular degeneration. Nat Rev Immunol.

[8] Wolff B, Macioce V, Vasseur V (2020). Ten-year outcomes of anti-vascular endothelial growth factor treatment for neovascular age-related macular disease: a single-centre French study [Epub ahead of print]. Clin Exp Ophthalmol.

[9] Alfadda AA, Sallam RM (2012). Reactive oxygen species in health and disease. J Biomed Biotechnol.

[10] Hsieh HL, Yang CM (2013). Role of redox signalling in neuroinflammation and neurodegenerative diseases. Biomed Res Int.

[11] McArdle A, Pollock N, Staunton CA, Jackson MJ (2019). Aberrant redox signalling and stress response in age-related muscle decline: role in inter- and intracellular signalling. Free Radic Biol Med.

[12] Winkler BS, Boulton ME, Gottsch JD, Sternberg P (1999). Oxidative damage and age-related macular degeneration. Mol Vis.

[13] Totsuka K, Ueta T, Uchida T (2019). Oxidative stress induces ferroptotic cell death in retinal pigment epithelial cells. Exp Eye Res.

[14] Ikwegbue PC, Masamba P, Oyinloye BE, Kappo AP (2017). Roles of heat shock proteins in apoptosis, oxidative stress, human inflammatory diseases, and cancer. Pharmaceuticals.

[15] Beere HM (2004). "The stress of dying": the role of heat shock proteins in the regulation of apoptosis. J Cell Sci.

[16] Cesa LC, Shao H, Srinivasan SR (2018). X-linked inhibitor of apoptosis protein (XIAP) is a client of heat shock protein 70 (HSP70) and a biomarker of its inhibition. J Biol Chem.

[17] Yang LL, Chen H, Wang J (2019). 4-HNE induces apoptosis of human retinal pigment epithelial cells by modifying HSP70. Curr Med Sci.

[18] Terman A, Gustafsson B, Brunk UT (2007). Autophagy, organelles and ageing. J Pathol.

[19] Cuervo AM, Dice JF (2000). Age-related decline in chaperone-mediated autophagy. J Biol Chem.

[20] Dice JF (2007). Chaperone-mediated autophagy. Autophagy.

[21] Richter K, Buchner J (2006). HSP90: twist and fold. Cell.

[22] Preissler S, Deuerling E (2012). Ribosome-associated chaperones as key players in proteostasis. Trends Biochem Sci.

[23] Mayer MP, Bukau B (2005). HSP70 chaperones: cellular functions and molecular mechanism. Cell Mol Life Sci.

[24] Ryhänen T, Mannermaa E, Oksala N (2008). Radicicol but not geldanamycin evokes oxidative stress response and efflux protein inhibition in ARPE-19 human retinal pigment epithelial cells. Eur J Pharmacol.

[25] Nillegoda NB, Kirstein J, Szlachcic A (2015). Crucial HSP70 co-chaperone complex unlocks metazoan protein disaggregation. Nature.

[26] Bascos NAD, Mayer MP, Bukau B, Landry SJ (2017). The HSP40 J-domain modulates HSP70 conformation and ATPase activity with a semi-elliptical spring. Protein Sci.

[27] Kopito RR (2000). Aggresomes, inclusion bodies and protein aggregation. Trends Cell Biol.

[28] Subrizi A, Toropainen E, Ramsay E, Airaksinen AJ, Kaarniranta K, Urtti A (2015). Oxidative stress protection by exogenous delivery of rhHSP70 chaperone to the retinal pigment epithelium (RPE), a possible therapeutic strategy against RPE degeneration. Pharm Res.

[29] Banjara M, Ghosh C (2017). Sterile neuroinflammation and strategies for therapeutic intervention. Int J Inflamm.

[30] Perkins ND (2007). Integrating cell-signalling pathways with NF-kappaB and IKK function. Nat Rev Mol Cell Biol.

[31] Kim JY, Yenari MA (2013). The immune-modulating properties of the heat shock proteins after brain injury. Anat Cell Biol.

[32] Dukay B, Csoboz B, Tóth ME (2019). Heat-shock proteins in neuroinflammation. Front Pharmacol.

[33] Krogh Nielsen M, Subhi Y, Molbech CR, Falk MK, Nissen MH, Sørensen TL (2019). Systemic levels of interleukin-6 correlate with progression rate of geographic atrophy secondary to age-related macular degeneration. Invest Ophthalmol Vis Sci.

[34] Yang HJ, Hu R, Sun H, Bo Chen, Li X, Chen JB (2019). 4-HNE induces pro-inflammatory cytokines of human retinal pigment epithelial cells by promoting extracellular efflux of HSP70. Exp Eye Res.

[35] Yu WW, Cao SN, Zang CX, Wang L, Yang HY, Bao XQ, Zhang D (2018). Heat shock protein 70 suppresses neuroinflammation induced by α-synuclein in astrocytes. Mol Cell Neurosci.

[36] Yang Y, Takeda A, Yoshimura T, Oshima Y, Sonoda KH, Ishibashi T (2013). IL-10 is significantly involved in HSP70-regulation of experimental subretinal fibrosis. PLoS One.

[37] Miller JW (2010). Treatment of age-related macular degeneration: beyond VEGF. Jpn J Ophthalmol.

[38] Zhang G, Liu Z, Ding H (2017). Tumor induces muscle wasting in mice through releasing extracellular HSP70 and HSP90. Nat Commun.

[39] Ryhänen T, Hyttinen JM, Kopitz J (2009). Crosstalk between HSP70 molecular chaperone, lysosomes and proteasomes in autophagy-mediated proteolysis in human retinal pigment epithelial cells. J Cell Mol Med.

[40] Lyu Q, Ludwig IS, Kooten PJS, Sijts AJAM, Rutten VPMG, van Eden W, Broere F (2020). Leucinostatin acts as a co-inducer for heat shock protein 70 in cultured canine retinal pigment epithelial cells. Version 2. Cell Stress Chaperones.

[41] Paimela T, Hyttinen JM, Viiri J, Ryhänen T, Karjalainen RO, Salminen A, Kaarniranta K (2011). Celastrol regulates innate immunity response via NF-κB and HSP70 in human retinal pigment epithelial cells. Pharmacol Res.

[42] Inagaki K, Shuo T, Katakura K, Ebihara N, Murakami A, Ohkoshi K (2015). Sublethal photothermal stimulation with a micropulse laser induces heat shock protein expression in ARPE-19 cells. J Ophthalmol.

[43] Wang J, Huie P, Dalal R (2016). Heat shock protein expression as guidance for the therapeutic window of retinal laser therapy. Proc SPIE.

